# The Impact of Experience, Length of Service, and Workplace Preparedness in Physicians’ Readiness in the Response to Disasters

**DOI:** 10.3390/jcm9103328

**Published:** 2020-10-16

**Authors:** Krzysztof Goniewicz, Mariusz Goniewicz, Frederick M. Burkle, Amir Khorram-Manesh

**Affiliations:** 1 Department of Aviation Security, Military University of Aviation, 08-521 Dęblin, Poland; 2 Department of Emergency Medicine, Medical University of Lublin, 20-059 Lublin, Poland; mariusz.goniewicz@umlub.pl; 3 Harvard Humanitarian Initiative, T.H. Chan School of Public Health, Harvard University, Boston, MA 02115, USA; fburkle@hsph.harvard.edu; 4 Department of Surgery, Institute of Clinical Sciences, Sahlgrenska Academy, Gothenburg University, 413 45 Gothenburg, Sweden; amir.khorram-manesh@surgery.gu.se; 5 Research Advisor, Department of Development and Research, Armed Forces Center for Defense Medicine, Gothenburg, 426 76 Västra Frölunda, Sweden

**Keywords:** emergency preparedness, physicians’ preparedness, disaster preparedness, hospital

## Abstract

With an increasing number of natural and man-made disasters, the need for preparedness in all levels of management is obvious. Among healthcare professionals responding to these emergencies, physicians are of particular importance due to their significant roles as leaders and frontline workers in minimizing morbidity and mortality of the affected population. This study analyses the preparedness of 549 physicians from all medical centers in Lublin, Poland to formulate their observations, suggestions, and recommendations concerning the improvement of the chain of response in disaster management. The results of this study show that the perceived preparedness of physicians for disaster management and response is not as high as it should be, and the majority of the respondents perceived their disaster preparedness insufficient. Training of physicians in disaster management and principles of disaster medicine is needed, by focusing on the specificity of rescue response to emergencies following disasters, and medical and non-medical aspects of the response with particular emphasis on a management approach covering all hazards.

## 1. Introduction

International statistics show that the frequency, magnitude, and significance of both natural and man-made disasters are constantly increasing. Every year, there are about 150 major disasters of different types worldwide, of which about 80% are floods and earthquakes. Among man-made disasters, fire incidents caused by deliberate arson or negligence concerning safety requirements dominate (about 70%). Fire disasters are associated with a high risk of serious injury and death. Equally tragic for people are disasters associated with the uncontrolled release of ionizing radiation or toxic chemicals [1]. The combination of terrorism and weapons of mass destruction is the greatest global threat [2]. Physicians play an integral role in the response to a disaster. Their role does not only encompass the provision of specialist medical assistance to the victims during the incident, but also the entire course of disaster preparedness [3,4]. The World Health Organization (WHO) defines a disaster as “an occurrence disrupting the normal conditions of existence and causing a level of suffering which exceeds the capacity of adjustment of the affected community” [5,6]. From a healthcare point of view, a disaster is an event where the number of affected people and medical problems exceeds the capabilities and capacities of the existing healthcare system. The main goal of medical rescue operations during disasters is to save the health and life of people in conditions which differ from everyday medical care by appropriate medical planning, organisation, logistics, and supply, as well as rescue tactics, triage, and treatment during a disaster, according to the principle of “what is best for the greatest possible number of victims, at the right time and in the right place” [7,8]. Many physicians are now required to obtain more specialist education and training in disaster medicine to be authorised to provide assistance in the event of a disaster [9]. This requirement results from medical, ethical, and legal issues related to healthcare dilemmas faced by physicians in times of limited resources and arising from extraordinary threats that occur as a result of a disaster, e.g., the coronavirus disease 2019 (COVID-19) pandemic [10]. At present, there are no strictly defined educational pathways in the field of disaster medicine [11]. There is no coherent, formalised training within and between institutions in Poland that would integrate training elements constituting the basis of disaster response [12]. As an example, in a community, every physician should prepare themselves, their families, and their patients for disasters. Such preparation should start with situational awareness, information sharing, and a disaster plan. Local and national plans should be available before occurrence of an unexpected event, and the continuous education of physicians in disaster management should be prioritised [13]. However, despite the significant role of physicians in responding to disasters, few studies directly measure aspects of their preparedness for these events [14]. To date, there has been no research conducted in Poland regarding the analysis of physician preparedness for disasters. This study is designed to understand the opinions of physicians working in Lublin concerning their role, knowledge, and experience with regards to disaster response. Understanding of the competences of physicians can be used to develop strategies for their effective use during disasters and further training.

## 2. Material and Method

### 2.1. Location of The Study

The study was conducted in May and June 2020 in the city of Lublin. It is the capital of the Lubelskie Province, the centre of the Lublin agglomeration, the ninth largest city in Poland in terms of population, and sixteenth largest in terms of area. Lublin is one of the most important and thriving academic centres in Poland, as well as a significant centre of primary and specialist healthcare. Lublin has several clinical hospitals, namely, two clinical hospitals of the Medical University, a university children’s hospital, and a military clinical hospital. In addition, the city also has a provincial specialist hospital, the Jan Bozy Hospital, the Centre of Oncology of the Lublin Region, a neuropsychiatric hospital, the Ministry (MSWiA) hospital, and the Institute of Rural Medicine. Education of future physicians is carried out in all hospitals. The Medical University in Lublin is the major centre for educating future medical staff in the region.

### 2.2. Study Population

Five hundred and forty-nine physicians from all hospital departments in all medical centres in Lublin were surveyed. The survey asked for a primary workplace, as many healthcare representatives work in several places.

### 2.3. Questionnaire

The initial questionnaire was developed based on a literature review by all authors. The following keywords were used: emergency preparedness; physicians’ preparedness; disaster preparedness; and the following search engines: PubMed, Scopus, and Web of Science. The acquired data were then organized, categorized, and mapped. Since the preparedness of health professionals for all threats is a comprehensive concept, in our study, we assessed the following elements of preparedness for all disasters that are likely to be reported in the regional crisis management plans: (a) perception of disaster risk, (b) experience of disaster response, (c) disaster training, and (d) preparedness for particular threats. The questionnaire (Supplementary Materials) consisted of 13 questions and was constructed to be completed in 5–10 min. There were five questions, which aimed to assess the perceived preparedness quantitively (questions 1–2, 7–9). Each question in this group was formulated as a statement, which could be answered using a Likert scale from 1 to 5, where 5 meant very well/high and 1 meant very low (very low, low, possible, probable, very well/high). Questions 3–6 dealt with experience, and could simply be answered by yes/no or by writing an option. The remaining questions 10–14 were demographic. To verify the research tool, the questionnaire was tested on a sample of 15 employees in one university hospital. This group was then excluded from the study and their answers were not included in the final analysis. The outcome was reviewed based on a combination of logic, relevance, comprehension, legibility, clarity, and usability.

### 2.4. Data Collection

Due to pandemic conditions, the survey was available both in paper form and online. The relevant authorities of the Medical University of Lublin were asked to disseminate the questionnaire in the online version. Five hundred and forty-nine physicians were provided with the survey, and all completed it.

### 2.5. Statistical Analysis

The statistical analyses were carried out with IBM SPSS Statistics version 23. It was used for frequency analysis and basic descriptive statistics. The classical statistical significance level was adopted as α = 0.05.

### 2.6. Ethical Considerations

The study is not a medical experiment and legally does not require the opinion of the Bioethics Committee. The participants received information about the study. The information included the study’s purpose, the voluntary nature of their participation, and strict confidentiality and secure data storage. It complied with the ethical principles stipulated by Polish law and thus was exempted from ethics approval requirements.

## 3. Results

The majority of respondents were men (54.3%). Similar values were recorded for all age ranges. Most physicians worked in a public hospital (79.8%). A majority of the respondents had been working for more than 20 years (35.5%), while 14.8% had up to 5 years of service. The results are presented in Table 1.

When asked about the probability of disasters occurring in Lublin in the next five years, the physicians indicated that epidemics and floods are possible, most probable, and of very high risk. A large group of respondents indicated that the occurrence of a large fire or drought is possible and probable. Quite a large group of respondents indicated the occurrence of a terrorist or bioterrorist attack, chemical, railway, and/or air disaster as possible. On the other hand, most of the respondents indicated the possible occurrence of an earthquake as low and very low. The results are presented in Table 2.

The respondents were then asked to indicate the place where they had provided assistance to victims of particular incidents. Most of the respondents had helped victims in two types of disasters: floods and epidemics. Out of the respondents who had helped flood victims, 66.5% had helped in a location other than Lublin. Of those who had helped victims of epidemics, most of them (89.1%) had helped in Lublin. In the case of other disasters, most respondents had helped victims in a location other than Lublin. The results are presented in Table 3.

Next, the respondents were asked to indicate what postgraduate training they had received. Most of the respondents (over 90%) had received first aid training, BLS (basic life support), and ALS (advanced life support). More than half of the respondents had participated in ACLS training (advanced cardiovascular life support) and triage. However, the vast majority of physicians stated that they had not received training in the following areas: psychological care (84%), crisis management (84.7%), humanitarian law (83.4%), or hazardous materials (HAZMAT) and chemical, biological, radiological, and nuclear (CBRN) (76.3%). The results are presented in Table 4.

**Figure 1:**
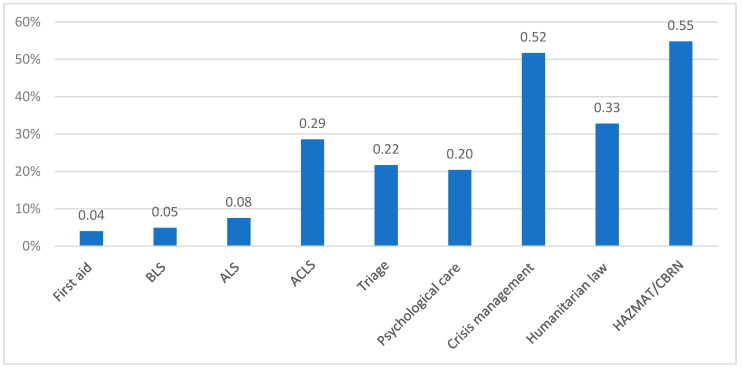
The distribution of preferences for specific training.

The respondents were then asked about their willingness to undergo additional disaster-related training. The most frequently indicated training courses were HAZMAT/CBRN (54.8%) and crisis management (51.7%). The other training courses were mentioned less frequently. The results are presented in Figure 1.

When asked whether their workplace offered disaster preparedness training and exercises, only 19.9% of the respondents stated that their workplace organised training and exercises connected with disaster preparedness.

**Figure 2:**
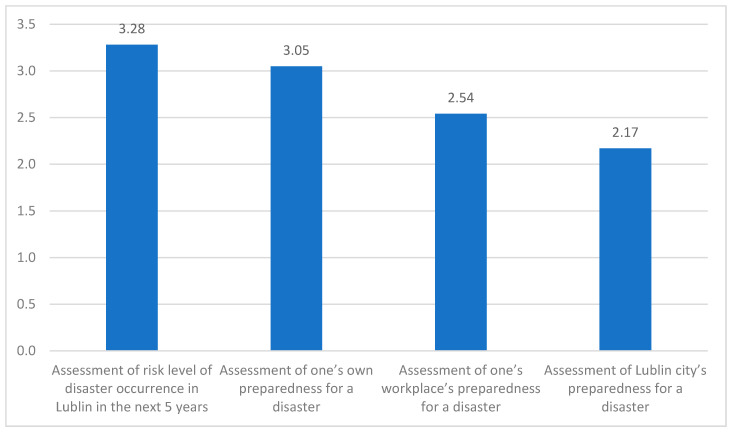
Averages for the assessment of risk level of disaster occurrence and preparedness for disasters. (Y axis = Likert scale from 0–5.).

In the following part of the survey, basic descriptive statistics were calculated for variables measured quantitatively to assess the individual perception of risk and disaster preparedness.

a. Participants ranked on a Likert scale from 0 to 5 the risk of a disaster occurring in Lublin within the next five years: the average risk was 3.28 (SD = 0.99). The median value for this variable was 3.00. The lowest value in the distribution was 1 and the highest was 5.

b. For the self-assessed perception of disaster preparedness, the average level of this variable was 3.05, with a deviation of ± 1.01. The median value for this variable was 3.00. The lowest value of the distribution was 1 and the highest was 5.

c. The mean value of the assessment of workplace preparedness for a disaster was 2.54, and the standard deviation was 0.85. The median value for the assessment of workplace preparedness for a disaster was 3.00. The lowest value chosen by the respondents was 1 and the highest was 4.

d. In regards to the assessment of Lublin’s disaster preparedness, the average level of the variable was 2.17, deviating ± o 0.78. The median value for this variable was 2.00. The lowest value recorded in the distribution was 1 and the highest 4 (Figure 2).

## 4. Discussion

The frequency of both natural and man-made disasters is increasing, causing significant economic, social, and environmental losses [15]. These trends have led to a change in the disaster management paradigm from reactive to proactive, i.e., new approaches to prevent a disaster and to minimise its potential outcomes, morbidity, and mortality [16]. Consequently, the proper response to disasters includes the early detection of threats and the implementation of measures in a timely manner [17]. Although the response to disasters and major incidents is a multi-agency task, and healthcare professionals represent only a small part of this collaborative approach, due to their significant role in emergency management, physicians’ preparedness to respond in such difficult circumstances is crucial [18].

The results of this study show that the perceived preparedness of physicians for disaster management and response is not as high as it should be, and the majority of the respondents perceived their disaster preparedness as insufficient [19,20]. One reason for such a perception may be the lack of education and the fact that disaster medicine is rarely included in pre- and postgraduate education in some countries such as Poland. Moreover, the lack of interest for the subject may result in an insufficient number of training opportunities and educational initiatives, and consequently many physicians have no opportunity to participate in disaster medicine courses designed to increase their competence and the level of required knowledge in disaster response. In this study, only 23.7% of physicians had completed a HAZMAT/CBRN course, and as many as 31.3% of physicians had not been trained in triage at all. In the current study, as many as 80% of the respondents reported that their workplace does not provide disaster medicine training. Since many physicians had not participated in rescue operations during a disaster or in the preparation of disaster response plans during their studies and professional practice, the nature of disasters makes it difficult for them to gain experience in everyday practice [19,20]. Therefore, continuous theoretical training in emergency response and emergency rescue exercises is necessary. The ability of physicians to understand and cooperate with local and regional emergency response systems is crucial in helping members of the public [21]. Therefore, courses and training in disaster medicine must include practical and theoretical information about the roles of each organisation in disaster response, particularly about agencies outside of the health sector [22,23]. However, it should be remembered that although multi-agencys’ exercises are an effective educational tool, they can be time- and resource-consuming and difficult to organise [24].

The axiom of disaster preparedness is that a successful disaster response is directly linked to pre- and post-event preparedness management. This study showed that physicians have a poor perception of their function and role in disaster response, which indicates a lack of preparedness. This is in line with the results of Carr et al.’s audit of doctors’ knowledge of major incident policies, in which less than 5% of the physicians surveyed were aware of their specific role in such an incident [25]. Furthermore, in this study, the participants reported a poor level of perceived disaster preparedness of their workplaces, which seems to be a key element, since according to our study, the level of workplace disaster preparedness has a considerable effect on the self-assessed perceived disaster preparedness of physicians. These findings are similar to those reported in previous reports, which showed inadequate preparedness for disasters in European hospitals [26,27,28]. A pilot study carried out by this group a few months earlier, covering one hospital in Lublin, also reported a low level of perceived preparedness for comprehensive crisis management [29]. Hospital managers should be particularly interested in these results and in finding an appropriate method at the operational level for distributing disaster policies and procedures to physicians and making sure they know and understand this important information. In this regard, the authority and responsibility of hospital managers must be clearly defined, understood, and standardised [30]. The current turnover of medical personnel is troublesome, and doctors are often burdened with a variety of roles. Hospitals are also not well organised or properly equipped, and do not have proper disaster procedures and equipment in place. A hospital’s disaster plan should be prepared before a disaster occurs. The basic provisions contained in a disaster plan should include coordination of the actions of all involved entities, detailed plans for the care of victims, and the training of all hospital staff in all disaster- and crisis-related hazards and areas [31]. Transparent procedures will ensure that action is effective in all phases, from the receipt of incident notification to action at the hospital ward level. Each emergency incident handled by the emergency medical system should be subject to extensive post-event analysis to define the advantages and disadvantages of the solutions used [32,33]. A thorough analysis of individual incidents reduces errors in subsequent rescue operations [34]. It is obvious that such a readiness cannot be gained during a disaster [35]. Djalali et al. have condemned the lack of education and training in disaster preparedness in health systems at the European Union level [36]. The promotion and improvement of training capacity in disaster medicine is one of the “call-to-action requirements” requested by the international community.

This study is the first analysis of physicians’ preparedness for disasters in Poland. It is also one of the few studies in Europe that provides valuable information on the perceived preparedness of physicians for mass casualty incidents. In future studies, the perceived level of disaster preparedness in a larger group of physicians across the country should be assessed, and the ways to increase the level of their disaster preparedness should be analysed and discussed.

### Limitations

The main limitation of this study is that it surveyed only a limited number of physicians from the city of Lublin. The COVID-19 pandemic proved to be an obstacle to further research, which could have impacted response rates and maybe generated response bias. There was also no subdivision between acute and critical care physicians, which will be included in further studies, as it is obvious that the variables studied will differ significantly between both groups. Despite these limitations, the study reveals gaps and training needs in terms of preparing physicians for disasters. It also opens up a discussion on this subject and the perspective of broader research in this area. The experience gained from this study will form the basis for planned future research. At the same time, it serves a wider standardisation of the research tool used.

## 5. Conclusions

Appropriate disaster response is a complex process that begins with risk assessment followed by risk management planning to prevent the occurrence of a threat. As one of the major actors in disaster response and management, physicians should be involved in risk assessment, planning, and training opportunities, where they can work before a disaster occurs, respond during a disaster, and help mitigate risks throughout the reconstruction period. Rescue operations during a disaster are extremely difficult and dangerous. Therefore, such rescue operations cannot be based solely on rigid procedures [37]. All actions and their management must be flexible to allow for a rapid response to changes in the situation. Only coordinated, properly planned, and practiced multi-agency actions can have real and measurable effects on the management of an emergency [38]. To prepare physicians for future disasters, it is mandatory to improve their educational opportunities in disaster medicine and disaster training. The responsibility for physicians’ preparedness should be overseen by decision makers at a local and national level [39]. The development of a standardised, approved education programme is an essential step in preparing not only physicians but all healthcare staff for proper disaster response.

## Figures and Tables

**Table 1:** Demographic data.

**Age**	***n***	**%**
Up to 34	127	23.1%
35–44 years	147	26.8%
45–54 years	134	24.4%
55 years and over	141	25.7%
**Gender**	*n*	%
Women	251	45.7%
Men	298	54.3%
**Length of service**	*n*	%
From 0 to 5 years	81	14.8%
6–10 years	89	16.2%
11–15 years	84	15.3%
16–20 years	100	18.2%
More than 20 years	195	35.5%
**Workplace**	*n*	%
Public hospital	438	79.8%
Research facility	110	20.0%
Outpatient Clinic	1	0.2%

**Table 2:** Likelihood of disasters occurring in Lublin in the next five years.

Risk of Disaster Occurring	Very low		Low		Possible		Probable		Very High	
Type of Incident	*n*	%	*n*	%	*n*	%	*n*	%	*n*	%
Flooding	19	3.5%	66	12.0%	136	24.8%	246	44.8%	82	14.9%
Epidemic	4	0.7%	31	5.6%	109	19.9%	243	44.3%	162	29.5%
Terrorist/bioterrorist attack	99	18.0%	176	32.1%	198	36.1%	76	13.8%	0	0.0%
Chemical disaster	67	12.2%	120	21.9%	249	45.4%	113	20.6%	0	0.0%
Air crash	82	14.9%	188	34.2%	208	37.9%	71	12.9%	0	0.0%
Railway crash	31	5.6%	136	24.8%	274	49.9%	104	18.9%	4	0.7%
Drought	5	0.9%	57	10.4%	221	40.3%	212	38.6%	54	9.8%
Large fire	17	3.1%	68	12.4%	217	39.5%	236	43.0%	11	2.0%
Earthquake	235	42.8%	198	36.1%	93	16.9%	23	4.2%	0	0.0%

**Table 3:** Experience in helping disaster victims.

Type of Incident	Place of Incident: Lublin		Other	
	*n*	%	*n*	%
Flooding	78	33.5%	155	66.5%
Epidemic	361	89.1%	44	10.9%
Terrorist/bioterrorist attack	0	0.0%	14	100.0%
Chemical disaster	1	2.2%	44	97.8%
Air crash	0	0.0%	14	100.0%
Railway crash	0	0.0%	38	100.0%
Drought	1	4.3%	22	95.7%
Large fire	14	17.7%	65	82.3%
Earthquake	1	3.4%	28	96.6%

**Table 4:** Training courses completed.

Type of Training	Yes		No	
	*n*	%	*n*	%
First aid	535	97.4%	14	2.6%
BLS	508	92.5%	41	7.5%
ALS	512	93.3%	37	6.7%
ACLS	349	63.6%	200	36.4%
Triage	377	68.7%	172	31.3%
Psychological care	88	16.0%	461	84.0%
Crisis management	84	15.3%	465	84.7%
Humanitarian law	91	16.6%	458	83.4%
HAZMAT/CBRN	130	23.7%	419	76.3%

## Supplementary Materials

The questionary used in this research is available online at https://www.mdpi.com/2077-0383/9/10/3328/s1, Rest of the datasets used and/or analysed during the current study are available from the corresponding author on reasonable request.
