# Supplementary material for: The Impact of Experience, Length of Service, and Workplace Preparedness in Physicians’ Readiness in the Response to Disasters
Source: J Clin Med. 2020 Oct 16;9(10):3328. doi: 10.3390/jcm9103328 (PMC7603037; doi:10.3390/jcm9103328)
Supplement: Supplementary file 1 [file jcm-09-03328-s001.pdf]

Appendix 1

Questionnaire: "Preparedness of doctors in the event of disasters"

Please read the questionnaire carefully and fill it in. When answering, please tick the appropriate boxes. The survey is completely anonymous. Your responses will only be used for statistical summaries.

1. **How do you assess the general level of risk of an occurrence in Lublin within the next 5 years, on a scale of 1 to 5?**

Very low risk 1 2 3 4 5 *Very high risk*

2. **How do you assess the likelihood of an event in Lublin within the next 5 years, on a scale of 1 to 5?**

(Where 1 is very low and 5 very high))

Flooding	1	2	3	4	5
Epidemic	1	2	3	4	5
Terroris / bioterrorist attack	1	2	3	4	5
Chemical disaster	1	2	3	4	5
Air Crash	1	2	3	4	5
Railway crash	1	2	3	4	5
Drought	1	2	3	4	5
Large Fire	1	2	3	4	5
Earthquake	1	2	3	4	5

3. **Have you helped victims of any of the following events, in Lublin or other place?**

	Lublin	Other Place
Flooding	☐	☐
Epidemic	☐	☐
Terrorist/bioterrorist attack	☐	☐
Chemical disaster	☐	☐
Air crash	☐	☐
Railway crash	☐	☐
Drought	☐	☐
Large fire	☐	☐
Earthquake	☐	☐

4. Have you received any of the following trainings?

	YES	NO
First Aid	☐	☐
ALS	☐	☐
BLS	☐	☐
ACLS	☐	☐
Triage	☐	☐
Psychological care	☐	☐
Crisis management	☐	☐
Humanitarian law	☐	☐
HAZMAT/CBRN	☐	☐

5. Which training would you like to participate?

a) First AID ☐
b) ALS ☐
c) BLS ☐
d) ACLS ☐
e) Triage ☐
f) Psychological care ☐
g) Crisis management ☐
h) Humanitarian law ☐
i) HAZMAT/CBRN ☐

6. Does the workplace offer trainings / exercises related to preparation for disaster?

- ☐ Yes
- ☐ No

7. How do you assess your own preparation for a disaster, on a scale of 1 to 5?

Very low 1 2 3 4 5 very well

8. How do you assess the current level of preparation of your workplace in case of a disaster, on a scale of 1 to 5?

Very low 1 2 3 4 5 very well

9. How do you assess the current level of preparation for the accident of the city of Lublin, on a scale of 1 to 5?

Very low 1 2 3 4 5 Very well

10. Gender:

- ☐ Men
- ☐ Women

11. Age:

- ☐ Up to 34
- ☐ 35-44
- ☐ 45-54
- ☐ 55 years and over

12. Workplace

- ☐ Public hospital
- ☐ Research facility
- ☐ Outpatient Clinic

13. Length of service:

- ☐ From 0 to 5 years
- ☐ 6-10
- ☐ 11-15
- ☐ 16-20
- ☐ More than 20 years

Thank you for completing the survey
